# Evaluating sources of technical variability in the mechano-node-pore sensing pipeline and their effect on the reproducibility of single-cell mechanical phenotyping

**DOI:** 10.1371/journal.pone.0258982

**Published:** 2021-10-25

**Authors:** Brian Li, Kristen L. Cotner, Nathaniel K. Liu, Stefan Hinz, Mark A. LaBarge, Lydia L. Sohn

**Affiliations:** 1 UC Berkeley–UCSF Graduate Program in Bioengineering, University of California, Berkeley and San Francisco, California, United States of America; 2 Department of Mechanical Engineering, University of California, Berkeley, California, United States of America; 3 Department of Population Sciences, Beckman Research Institute, City of Hope, Duarte, California, United States of America; Texas A&M University College Station, UNITED STATES

## Abstract

Cellular mechanical properties can reveal physiologically relevant characteristics in many cell types, and several groups have developed microfluidics-based platforms to perform high-throughput single-cell mechanical testing. However, prior work has performed only limited characterization of these platforms’ technical variability and reproducibility. Here, we evaluate the repeatability performance of mechano-node-pore sensing, a single-cell mechanical phenotyping platform developed by our research group. We measured the degree to which device-to-device variability and semi-manual data processing affected this platform’s measurements of single-cell mechanical properties. We demonstrated high repeatability across the entire technology pipeline even for novice users. We then compared results from identical mechano-node-pore sensing experiments performed by researchers in two different laboratories with different analytical instruments, demonstrating that the mechanical testing results from these two locations are in agreement. Our findings quantify the expectation of technical variability in mechano-node-pore sensing even in minimally experienced hands. Most importantly, we find that the repeatability performance we measured is fully sufficient for interpreting biologically relevant single-cell mechanical measurements with high confidence.

## Introduction

**Fig 1:**
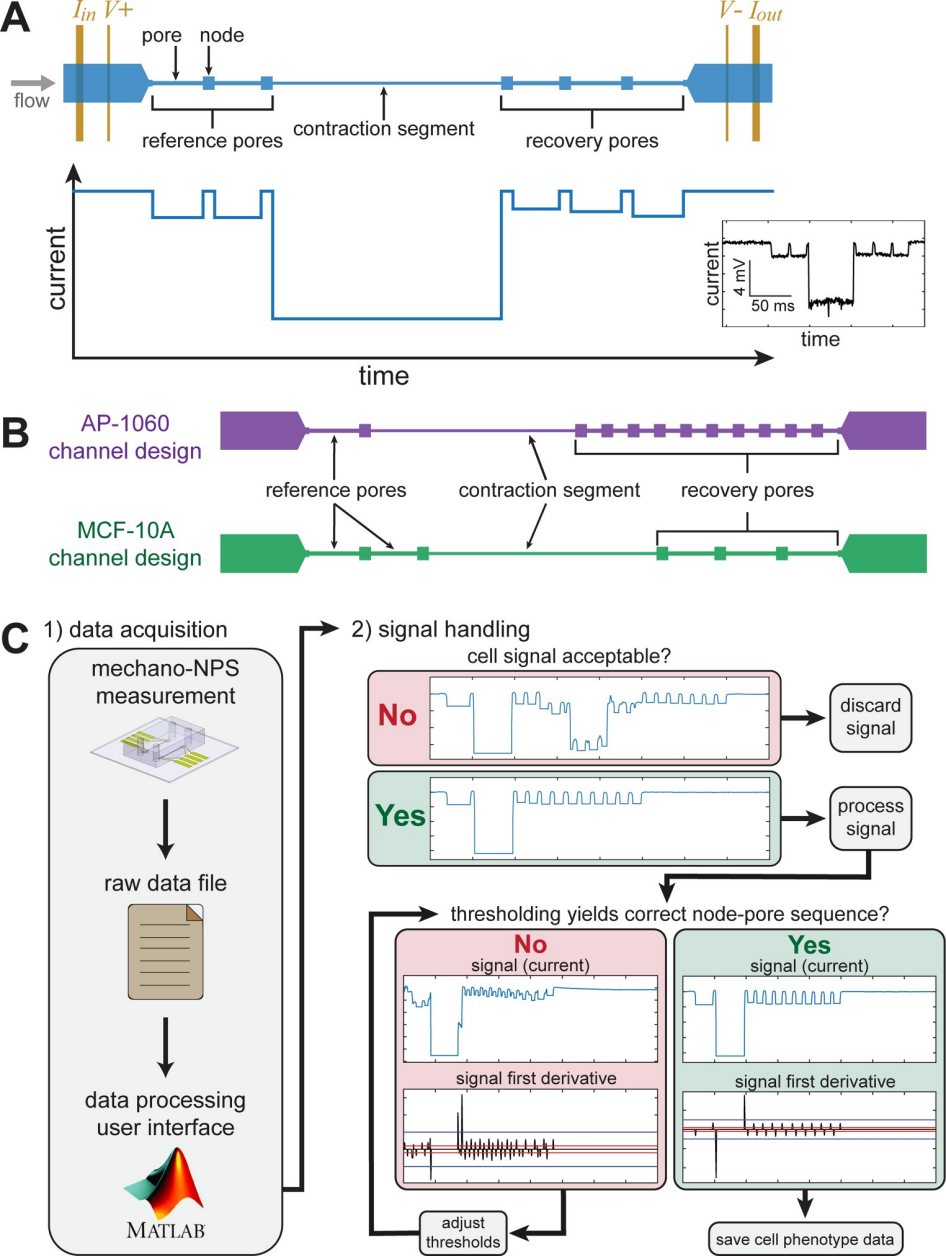
Overview of mechano-node-pore sensing (mechano-NPS) operating principles, device design, and data processing pipeline. (A) Top-down schematic view of a mechano-NPS device (top), with corresponding expected electric current pulse (bottom) caused by a single cell transiting the microfluidic channel. A potential is applied across the channel, causing a drop in measured current when a cell enters a narrow segment of the microchannel (pore). Inset: An actual current pulse caused by an MCF-10A cell traversing the channel. (B) Top-down schematic views of mechano-NPS channel designs used in this work for screening AP-1060 and MCF-10A cells. (C) Flow of user input to the data processing analysis software. The user must identify valid signals corresponding to cell measurements and exclude non-valid pulses. Subsequently, the user must set processing thresholds to the appropriate levels for each valid cell.

As cells frequently generate and experience a variety of forces in normal physiology, their mechanical properties are an important aspect of their function. Cell mechanical properties are implicated in many diseases, including metastatic cancer and a variety of laminopathies [1, 2]. More recently, there has been increasing research on the use of “mechanical phenotyping” to identify and screen single cells for mechanical properties associated with malignancies [3–5]. In this work, we focus specifically on the use of mechano-node-pore sensing (mechano-NPS) to analyze cells from breast epithelial tissue and from a drug-resistant leukemia cell line [5, 6]. To extract mechanical information from cells, mechano-NPS uses a four-terminal measurement across a microchannel to track cell transit times as they are deformed by a narrow constriction [7, 8] (Fig 1A). Mechano-NPS, like other microfluidic methods for single-cell mechanical testing, has demonstrated enhanced deformability in cancer cells, reflecting invasive potential [1, 3, 5, 9, 10]. Uniquely, by measuring cell recovery from deformation, mechano-NPS has also been used to uncover age-dependent changes in viscoelastic properties and offers roughly 10-fold faster throughput for viscoelastic testing over more established methods such as optical tweezers [5, 11].

Previously, the technical variability of microfluidic techniques like mechano-NPS has only been addressed through simple validation and calibration. For mechano-NPS, Kim et al. made comparisons to published measurements of cortical tension and elastic modulus for several cell lines, showing that their measure of deformability followed trends established with atomic force microscopy and micropipette aspiration, the gold standards for measuring cell mechanical properties [5]. Similarly, a comparison among three recently developed mechanophenotyping microfluidic techniques—hydrodynamic stretching, suspended microchannel resonators, and real-time deformability cytometry—show that all three can, with limited agreement, sense similar trends in deformability [12]. To calibrate hydrodynamic stretching, Gossett et al. carried out measurements on droplets of various viscosities, demonstrating the relationship between measured deformability and known values of viscosity [13]. Kang et al. calibrated their suspended microchannel resonators using polystyrene beads and hydrogel spheres of varying elastic modulus [14]. For real-time deformability cytometry (RT-DC), Mietke et al. and Girardo et al. employed theoretical modeling of soft matter deformation in combination with experimental measurements of agar and polyacrylamide beads of varying stiffnesses to validate observed changes in apparent cell deformability as measured by RT-DC [15, 16]. Notably, only Gossett et al. and Kim et al. performed any reliability testing of their respective platforms, and even here, such testing was limited to assessing only a handful of sources of variability [5, 13]. It is thus clear that the field is lacking in reproducibility analyses for microfluidic platforms that measure single-cell mechanical properties. Such analysis would provide a critical performance benchmark for the growing number of researchers who may seek to adopt these technologies for their own applications.

Observing this need for reproducibility analyses in our field, we set out to examine the reproducibility of the mechano-NPS system and its measurements of single-cell mechanical phenotypes. First, to characterize device-to-device variability, we quantified the differences in mechano-NPS results when a single biological sample is tested across multiple replicate devices with relatively low sample sizes. Next, we examined the intra- and inter-user reliability of the semi-manual mechano-NPS data processing pipeline by analyzing results obtained by several researchers using our MATLAB command-line interface (CLI) [6] to process identical mechano-NPS raw data sets. This CLI replaces a time-consuming, fully manual data processing pipeline that took, at minimum, one minute to find and analyze a cell. In contrast, our new CLI is capable of analyzing up to ~10 cells per minute. Thus, we evaluated whether this new software could generate variable results based on user input. Last, to assess the reproducibility of the mechano-NPS technology platform, we evaluated the similarity in results from two identical experiments conducted on two different sets of mechano-NPS hardware by different researchers in different physical locations.

Overall, we show that average measures of single-cell mechanical parameters using mechano-NPS are highly repeatable. Using our new CLI, both experienced and novice users were able to process hundreds of cell events rapidly and produce highly consistent results. We show that the current mechano-NPS analysis pipeline is capable of high degrees of consistency, high throughput (allowing for large sample sizes), and significantly faster analysis of large data sets compared to prior, manual methods. This work provides an important insight into the performance and reproducibility of mechano-NPS, demonstrating its maturity as a technology and potential for further adoption by other research groups.

## Results and discussion

### Mechano-NPS principles, device design, and testing procedures

In node-pore sensing (NPS), a microfluidic channel is segmented into wider “nodes” and narrower “pores.” As cells flow through the channel, unique current (or resistive) pulses are measured using a four-terminal measurement (Fig 1A). Each pulse consists of subpulses that are generated when a cell transits the pores within the channel and partially blocks the flow of current. The magnitude of a subpulse corresponds to the size of the cell, and the duration corresponds to the transit time of the cell. In mechano-NPS, the channel is designed such that a cell first passes through reference pore(s) and then through a narrow “contraction” segment designed to apply a specific strain (defined as the percent-change in cell diameter along the axis of deformation). Reference pore(s) measure the cell’s initial diameter and velocity, and the contraction segment measures the cell’s resistance to deformation. After passing through the contraction segment, the cell transits several “recovery” pores, which measure the cell’s relaxation to its original size and shape after deformation.

The geometry of each mechano-NPS device is optimized to measure a particular cell size. To assess the reproducibility of previous studies using mechano-NPS, we employed the same device designs from Kim et al. and Li et al. to measure MCF-10A and AP-1060 cells, respectively [5, 6] (Fig 1B). Both devices assess a cell’s elastic deformability according to its transit time through the contraction segment using a unitless number referred to as the whole-cell deformability index (*wCDI*):

$$wCDI=(v_{c}/v_{0})(d_{0}/h),$$ (1)

where *v*_*c*_ is the cell’s velocity in the contraction segment, *v*_*0*_ is the cell’s velocity in the reference pore, *d*_*0*_ is the cell’s initial diameter, and *h* is the height of the channel. As shown by Kim et al., a cell’s *wCDI* is inversely related to its Young’s modulus [5]. While *wCDI* characterizes the cell’s elastic properties, the cell’s viscoelastic behavior is assessed by measuring its recovery from the deformation applied in the contraction segment. For MCF-10A cells measured with the device design described by Kim et al., cells are classified into distinct recovery categories defined by how long the cell took to return to its original shape after deformation. For AP-1060 cells measured with the device design described by Li et al., a quantitative recovery time constant (*τ*) is calculated from the rate at which the cell relaxes from a deformed ellipsoid to a sphere. We first evaluated how device-to-device variability and our data processing pipeline (Fig 1C) affect mechano-NPS reproducibility, using AP-1060 cells as an example. We then assessed the overall reproducibility of mechano-NPS measurements in two different laboratories using identical devices and MCF-10A cells from the same frozen source.

### Device-to-device variability and its effects on mechanical phenotyping

We first characterized how device-to-device variability could affect mechano-NPS reproducibility, which can be masked when pooling data from several replicate devices and samples. Device-to-device variability can arise from manufacturing, resulting in differences in channel geometry (e.g., channel height and width) that in turn may affect flow rates through a given device. We mechanically phenotyped small samples of AP-1060 cells (127 ≤ *n* ≤ 184), determining the *wCDI* and recovery time constant using seven different mechano-NPS devices as described by Li et al. [6] (Fig 1B).

**Fig 2:**
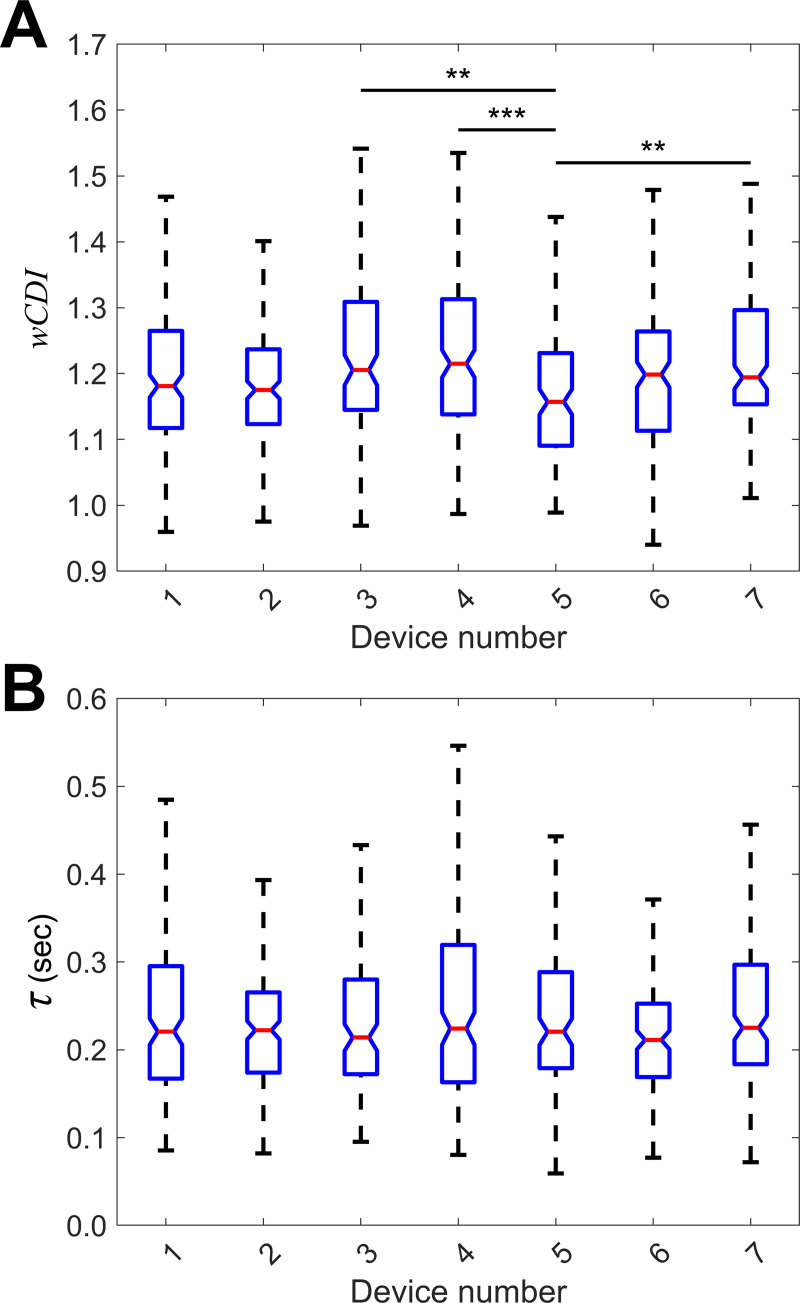
Between-device variability of mechanical phenotyping parameters measured by mechano-NPS. (A) Box plots of whole-cell deformability index (*wCDI*) for a single sample of AP-1060 cells analyzed using seven separate devices. Notches represent 95% confidence intervals for the true sample median for each distribution. ** indicates *p <* 0.01; *** indicates *p* < 0.001. Statistical significance between devices was determined using pairwise Wilcoxon rank sum tests for unequal medians, where the critical value for multiple comparisons was calculated using a Tukey-Kramer method. (*p*_3,5_ = 0.0021, *p*_4,5_ = 0.0007, *p*_5,7_ = 0.0010; *n*_1_ = 182, *n*_2_ = 184, *n*_3_ = 127, *n*_4_ = 176, *n*_5_ = 134, *n*_6_ = 155, *n*_7_ = 167). (B) Box plots of recovery time constant (*τ*) for the AP-1060 cells analyzed in (A) on each of seven devices. Notches represent 95% confidence intervals for the true sample median for each distribution. A Kruskal-Wallis test failed to reject the null hypothesis that the recovery time constants measured by each device came from the same distribution. (*n*_1_ = 182, *n*_2_ = 184, *n*_3_ = 127, *n*_4_ = 176, *n*_5_ = 134, *n*_6_ = 155, *n*_7_ = 167).

We found the *wCDI* distributions (Fig 2A) from each device to be non-normal (S1 Table). We tested whether the data from these seven devices were sampled from the same distribution with a Kruskal-Wallis test and found that these data reject the null hypothesis of an equal originating distribution (*p* < 0.0001). Pairwise comparisons for all devices showed that only Device 5was significantly different from Device 3 (*p =* 0.0021), Device 4 (*p* = 0.0007), and Device 7 (*p* = 0.0010) (Fig 2A). Pairwise tests of *wCDI* cumulative distribution functions (CDFs) similarly showed that the *wCDI* data from Device 5 was not sampled from the same distribution as Device 3 (*p* = 0.0012), Device 4 (*p* = 0.0004), or Device 7 (*p* < 0.0001) (Bonferroni-corrected for 21 pairwise comparisons, *α* = 0.0024), whereas all other *wCDI* data from each device were sampled from the same distribution (S2 Table).

We performed a similar analysis on recovery time constant data from the same cells (Fig 2B). We found that the distributions from several devices were non-normal (S1 Table). Analyzing the recovery data obtained from these seven devices, we found that the recovery time constant data for all seven groups were sampled from the same distribution (*p =* 0.21), precluding the need for further pairwise comparisons. This analysis suggests that compared to *wCDI*, the measurement of continuous recovery time constant is less sensitive to device-to-device variability and is thus more robust against variability in manufacturing.

For typical mechano-NPS experiments, the data from replicate devices are pooled before comparisons are made among experimental conditions [5, 6]. The discrepancy among *wCDI* data from the different devices highlights the importance of this data pooling, which reduces the influence of device-to-device variability. This variability may cause the *wCDI* for cells in a certain condition or sample to appear more extreme than they truly are, whereas combining the measurements from several devices increases the statistical power of tests against other conditions by reducing the influence of such extreme values to the test statistic at hand. Even though similar variations were not observed in recovery time constant measurements, data pooling would serve the same purpose. In comparing the difference in median *wCDI* among the seven devices, there was only a 5.04% difference in the two most extreme median values (between Device 5 and Device 6). Devices 3 and 4, which were the next most extreme pair, only varied in median *wCDI* by 2.46%. We consider even 5% variance to be within acceptable tolerances for mechano-NPS devices, as they account for a difference in *wCDI* of approximately 0.06, whereas biologically meaningful differences in *wCDI* using this device design often exceeded 0.1 [6]. While biological variability may also influence this result, each of the seven samples of AP-1060 cells were biological replicates and were handled identically prior to mechano-NPS measurements (see Methods). While this served to minimize differences between replicates, biological variability is impossible to completely eliminate. As such, the measured 5.04% difference in median *wCDI* may be an overestimate of the actual device-to-device variability in mechano-NPS. Overall, we demonstrate the degree to which device-to-device variability in mechano-NPS can affect measurements of *wCDI* and recovery time constant.

### Intra- and inter-user reliability of results obtained using mechano-NPS data processing pipeline

**Fig 3:**
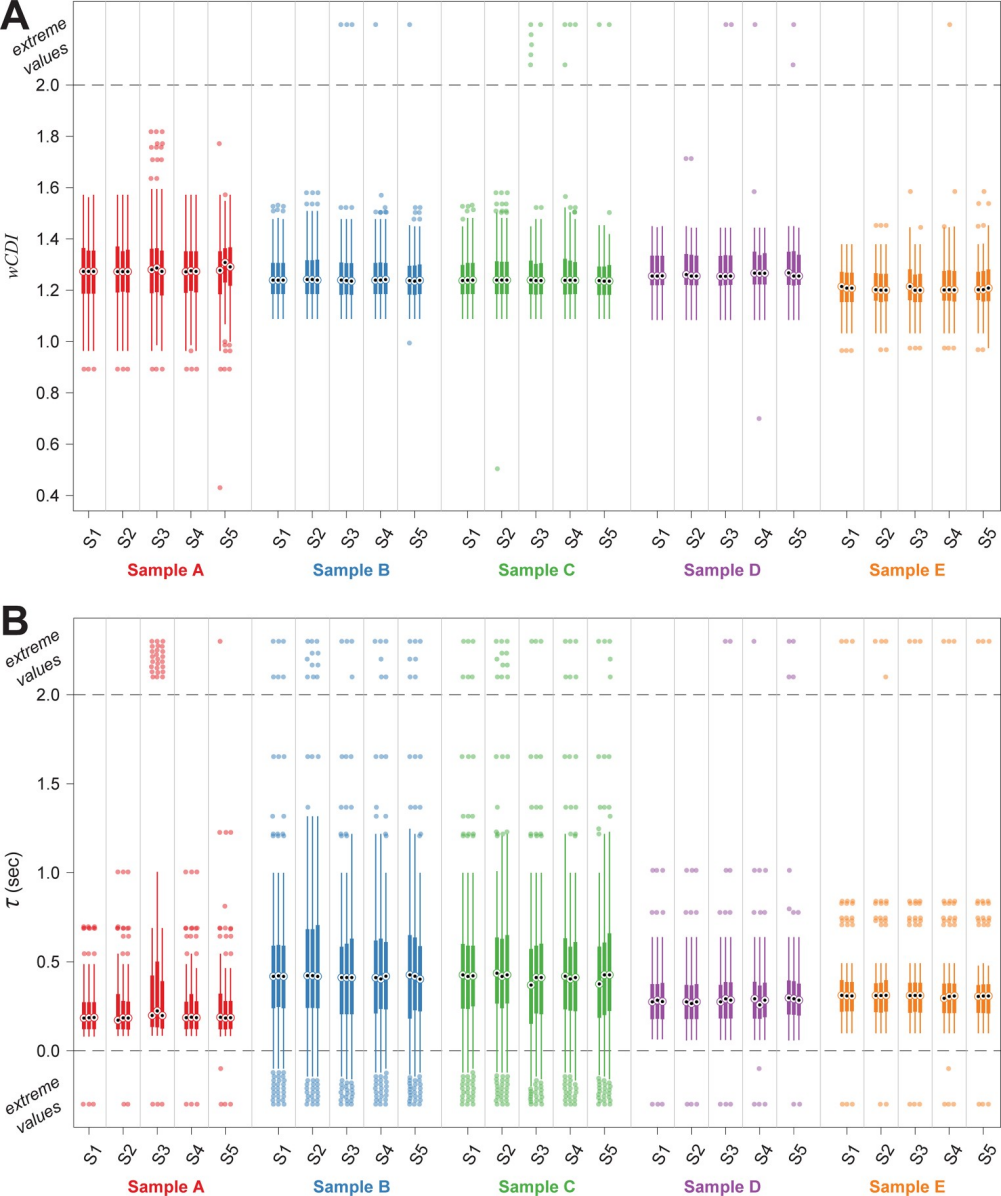
Intra- and inter-user comparison of results obtained using the mechano-NPS data processing pipeline. Processed data obtained from measurements of AP-1060 cells. Data sets A–C were obtained from measuring untreated cells, while data sets D–E were obtained from measuring cells treated with Latrunculin A (LatA). Plots show *wCDI* (A) and recovery time constant *τ* (B) results, respectively. Processed data is shown from each of the five subjects across three replicate data processing tasks. Data is presented as originally returned after the data processing task was completed (i.e., before erroneous measurements were excluded). Sample medians are represented by black dots; sample interquartile ranges are represented by thick lines; outliers are represented by filled circles and are defined as 1.5 times the inter-quartile range. Extreme values are represented as filled circles placed above or below the dashed lines and are defined as >2 or <0 for both *wCDI* and *τ*. Number of cells found in each observation ranged from 49–82.

As the processing of mechano-NPS data is another critical aspect for producing consistent results, we quantified the variability introduced through the semi-manual nature of our custom mechano-NPS data processing software [6] (Fig 1C). We recruited five subjects who are microfluidics researchers with varying degrees of familiarity with mechano-NPS and surveyed them about their familiarity with the mechano-NPS data processing software. Subjects 1–2 identified as experienced users of the software, while Subjects 3–5 identified as novice users of the software. We then provided all subjects with a copy of the mechano-NPS data processing software and a 30-minute training video on how to use it. All subjects were tasked with performing data processing on five blinded mechano-NPS raw data sets using the custom software. These data sets comprised five measurements (A–E) of AP-1060 cells (Fig 3, S1 Fig). Data sets A–C were obtained from measuring untreated cells, while data sets D–E were obtained from measuring cells treated with the microfilament-disrupting agent Latrunculin A (LatA). Additionally, each subject performed analysis on each data set three different times in order to test intra-user reliability. Overall, we examined both intra- and inter-user reliability using the results from experienced and novice users of the software.

The data processing software asks for user input at two different stages for each potential cell measurement (Fig 1C): First, the user must decide whether to save the cell measurement or to discard it (in cases where the presented measurement is not a valid cell pulse, or when cell measurements overlap). If the user decides to save the cell, they are then asked to set two thresholds that affect the cell phenotype measurements that are recorded (see Methods section for additional details on the data processing pipeline). Since these data sets were obtained using the AP-1060 device design, there are two continuously distributed mechanical phenotyping measurements recorded for each cell: *wCDI* and recovery time constant *τ*. The number of cells identified by a single user from a single raw data file ranged from 49–82.

**Fig 4:**
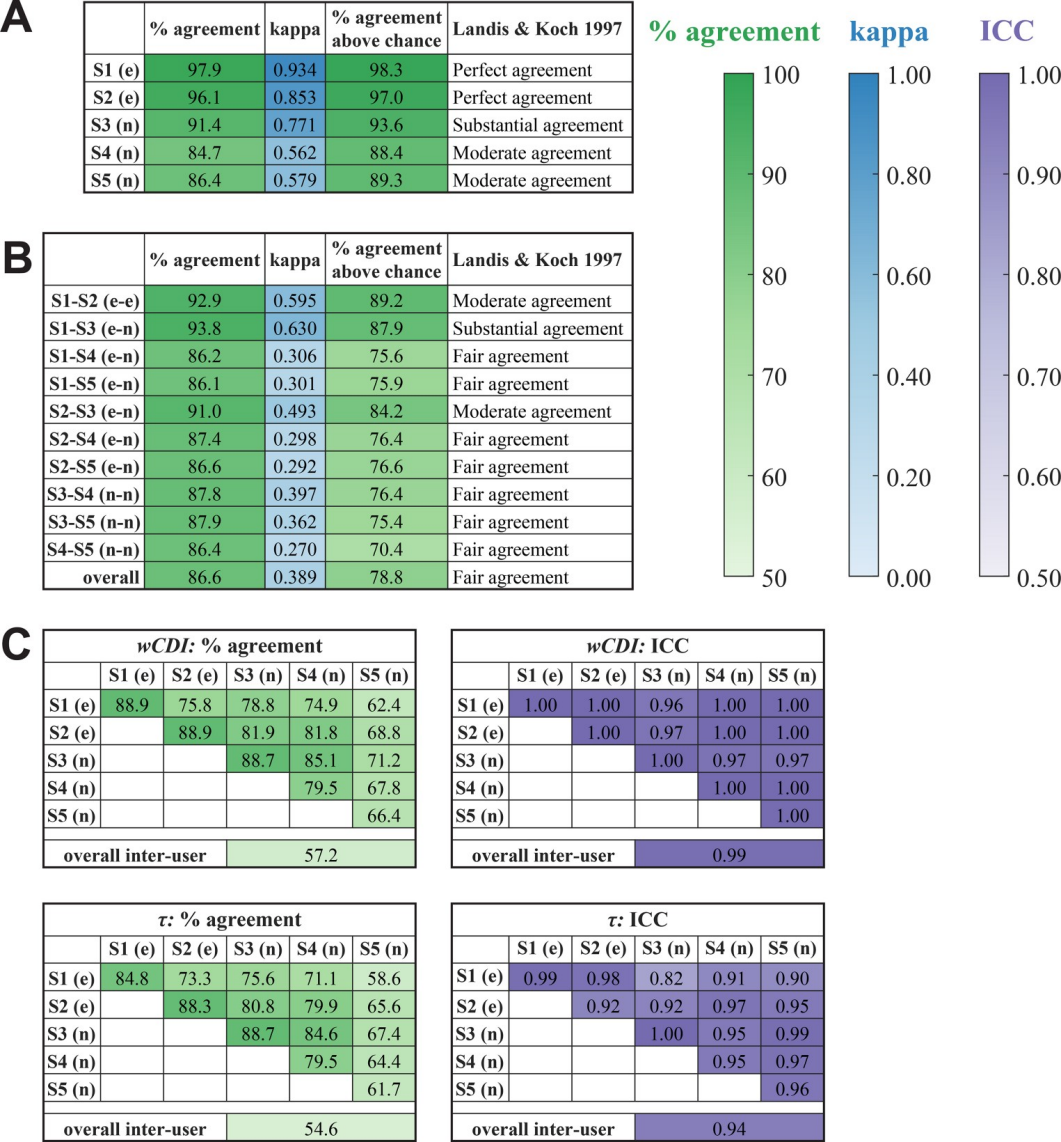
Intra- and inter-user reliability analysis of the mechano-NPS data processing pipeline. Reliability analysis results obtained from the data presented in Fig 3 (e) and (n) designate experienced and novice users, respectively. Number of cells found in each observation ranged from 49–82. Intra- (A) and inter-user (B) agreement analysis examines the consistency of users’ decisions to save or discard cell measurements. “% agreement” quantifies the percentage of potential cell events in which all observations agreed on whether to save or discard the event. Fleiss’s kappa analysis was also performed to determine the “kappa” value as well as the “% agreement above chance.” “Landis & Koch 1997” indicates the interpretation of the kappa value according to [17]. For all comparisons, Fleiss’s kappa analysis rejected the null hypothesis that the observed agreement was accidental (*p <* 10^−10^). (C) Intra- and inter-user concordance analysis examines the consistency of the observed quantitative cell phenotypes for each of the two phenotype variables: *wCDI* and recovery time constant *τ*. “% agreement” quantifies the percentage of cell events in which all observations found the same phenotype value, within tolerance. We also calculated the intra-class correlation (ICC) value, which quantifies the degree of correlation among the observations. For all comparisons, ICC analysis rejected that the null hypothesis that *ICC* = 0 (*p <* 10^−10^).

We first analyzed the consistency of each user’s decisions to save or discard cell measurements. We calculated percent-agreement and performed Fleiss’s kappa analysis on the decision to save or skip each cell. The intra-user agreement analysis (Fig 4A) shows that all subjects exhibited a high degree of self-consistency in their decisions to save or discard cell measurements. The experienced software users were designated as showing “perfect agreement” according to Landis & Koch’s interpretation of the Fleiss’s kappa values [17], while the novice users demonstrated “substantial” or “moderate” agreement. For all users, the null hypothesis was rejected in Fleiss’s kappa analysis, indicating that the observed agreement was not accidental. The inter-user Fleiss’s kappa analysis (Fig 4B) shows that the overall consistency between all subjects was moderately high (~80% agreement above chance), and “fair agreement” was observed. Pairwise analysis of the agreement between subjects reveals that the experienced users and one novice user (Subject 3) had a high degree of agreement with each other. Subjects 1‍–‍3 showed “moderate” or “substantial” agreement with each other, while all other subjects showed “fair agreement” with each other. For all comparisons, the null hypothesis was rejected. Importantly, Fleiss may be an overly conservative measure of agreement because it considers the possibility that users may assign labels randomly, which is unlikely in this data processing task. Moreover, this analysis cannot account for agreement on cells that were skipped in all observations, so the probability of encountering a cell that should be saved (and thus, the probability of agreeing to save a cell by chance) is highly overestimated in the Fleiss calculation. For these reasons, we have also calculated the raw percent-agreement value, which directly quantifies the percentage of times that the observations agreed on whether to save or skip a cell. For all intra- and inter-user analyses, the raw percent-agreement was ~85% or higher, and the two experienced users and Subject 3 showed >90% raw agreement among themselves.

We then analyzed the consistency of the observed quantitative cell phenotypes, which can be affected by the thresholds set by the user. For each of the two mechanical phenotyping variables, we calculated the percentage of cells where an equivalent value was found in each observation, and we also calculated the intra-class correlation (ICC) to quantify the correlation in the measured values between observations. The intra-user analysis (Fig 4C, along the diagonals) shows that all subjects found equivalent values for both variables a majority of the time; most of the users found equivalent values for both variables ~80% of the time or more, although one of the novice users (Subject 5) only found equivalent values for the variables ~60–70% of the time. However, the intra-user correlation of values was very high for all subjects (*ICC* > 0.9), demonstrating a very high degree of self-consistency in both variables. The inter-user analysis (Fig 4C) was similar to the intra-user analysis, showing that subjects found equivalent values with each other for both *wCDI* and *τ* a majority of the time. We again find that the two experienced users and one of the novice users (Subject 3) showed a higher degree of consistency among themselves than with the other two novice users according to the percent-agreement analysis. However, the ICC analysis showed a very high degree of correlation in both variables among all users (*ICC* > 0.9 in all but one comparison). For all intra-and inter-user analyses, the null hypothesis that *ICC* = 0 was rejected for both *wCDI* and *τ*.

Overall, the agreement and correlation analyses demonstrate that the semi-manual mechano-NPS data processing platform enables reproducible results. Users demonstrate consistent results upon repeated analysis of the same data set, and different users find results that are consistent with each other. Although experienced users do show a higher degree of consistency than novice users, novice users of this software are still able to achieve reliable results right away. This finding is critical for ensuring that the mechano-NPS platform, and its associated data processing pipeline, are reproducible even when adopted by new and inexperienced users.

### Comparing mechano-NPS results from experiments performed on different instrumentation platforms

Finally, we investigated whether results from a mechano-NPS experiment are reproducible if another research group utilizes a physically different mechano-NPS platform and conducts the same experiment. Two mechano-NPS platforms were used in this comparison: one located in Berkeley, California and operated by mechano-NPS researchers (Site A), and another located in Duarte, California and operated by a collaborating research group specializing in breast cancer research (Site B) (see Methods for a complete list of hardware). Although there were slight differences in the instrumentation used (e.g., digital vs. analog current preamplifier), the platforms are functionally identical. At both sites, an identical mechano-NPS device design was used to measure MCF-10A cells (Fig 1B), and all devices were fabricated at Site A using an identical process (see Methods) [5]. We chose to measure MCF-10A cells in this experiment instead of AP-1060 cells (as above) due to the expertise in breast cancer at Site B (and by extension, the availability of devices designed to measure breast epithelial cells). To compare mechanical phenotyping results, replicate vials of cryo-preserved MCF-10A cells were distributed from Site B, thawed, and cultured in identical growth medium (see Methods). A researcher at each site measured the cells using that site’s mechano-NPS platform and extracted two mechanical phenotyping parameters—cell deformability and recovery category—as described in previously published work (Fig 1) [5].

**Fig 5:**
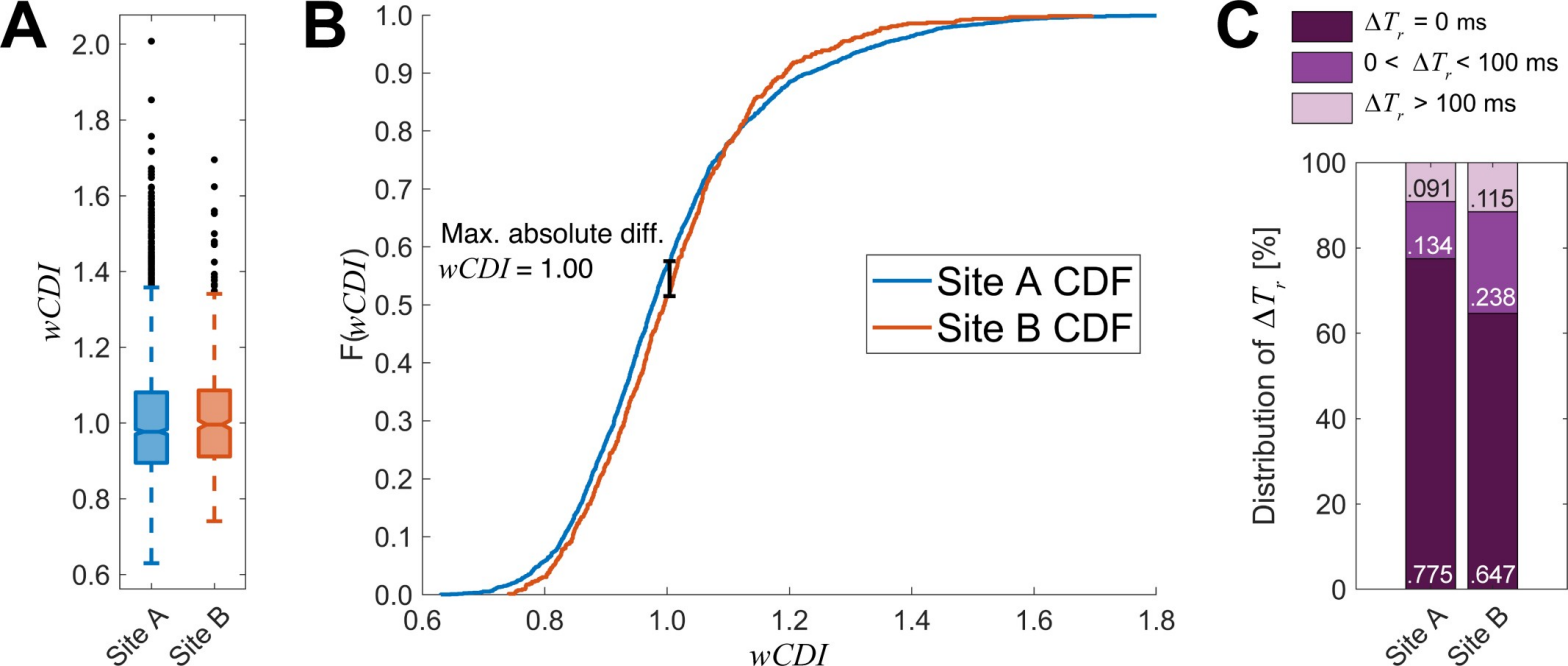
Mechano-NPS results from different instrumentation sets at location Sites A and B are highly reproducible. (A) Box plots of *wCDI* for MCF-10A cells analyzed at Site A and Site B. Black points represent cells with outlier values of *wCDI*, defined as 1.5 times the inter-quartile range. Notches represent 95% confidence intervals for the true sample median for each distribution. (Site A *n =* 1960, Site B *n =* 625). (B) Empirical CDFs for Site A and Site B *wCDI* data. A two-sample Kolmogorov-Smirnov test determined that these data are sampled from different distributions (*p =* 0.042; Site A *n =* 1960, Site B *n =* 625), with the maximum absolute difference occurring at *wCDI =* 1.00. (C) Stacked bar graph representing the relative categorical frequency of MCF-10A cells recovering instantaneously (*ΔT*_*r*_ = 0 ms), within the finite time window (0 < *ΔT*_*r*_ < 100 ms), or failing to recover within the finite time window (*ΔT*_*r*_ > 100 ms). Chi-squared analysis determined that these frequencies were statistically significantly different (*p <* 0.0001; Site A *n* = 1960, Site B *n* = 625; see S6 Table).

Between Site A and Site B results, the MCF-10A mean and median *wCDI* varied by less than 0.4% and 2%, respectively (Fig 5A). Lilliefors tests for *wCDI* distributions determined that both Site A (*p <* 0.001) and Site B (*p <* 0.001) distributions were non-normal, and a Mann-Whitney U-test found that the median *wCDI*s were not statistically significantly different (*p =* 0.055). We then performed a post hoc power analysis of this test to determine the minimum effect size detectable with 80% power and a significance criterion of *α* = 0.05. We computed this effect size to be 0.0275; since the actual effect size for this experiment was only 0.0201, we conclude that the difference in median *wCDI* between Site A and Site B is not a meaningful difference. However, we compared the empirical CDFs for *wCDI* data obtained at Site A and Site B (Fig 5B) and found that they were statistically significantly different (*p =* 0.042), with the maximum absolute deviation occurring between the sample medians.

We then investigated the reproducibility of MCF-10A recovery categories. Kim et al. classified cell recovery into three categories: immediate, finite, and prolonged. A cell is classified as “immediate” if it is observed to recover its original shape immediately, “finite” if it recovers within a finite time range, or “prolonged” if it does not recover within the finite range (implying slow, prolonged recovery) [5]. We classified the MCF-10A recovery categories measured by Site A and Site B and, using a Pearson’s Chi-squared test, found that the frequencies of recovery categories were significantly different (*p <* 0.0001) with a Cramér’s V of 0.094, which is considered a small-to-medium effect size (Fig 5C) [18–20].

By comparing *wCDI* and recovery category data between the two sites, we observed statistically significant differences according to a standard significance criterion of 0.05. As mechano-NPS is easily capable of measuring hundreds to thousands of cells, it is reasonable that even small differences may be statistically significant. In this case, we would expect differences in cell culturing and handling (e.g., expert vs. novice tissue culture technique, lot-to-lot differences in trypsin-based disassociation solutions, etc.) to account for some differences in measured mechanical properties such as those observed here. Furthermore, based on our characterization of device-to-device variability, we cannot exclude the possibility that manufacturing variations also contribute to the differences observed between Site A and Site B.

The effect sizes we observed between sites are small in comparison to biologically meaningful effect sizes observed in past mechano-NPS work. For example, Kim et al. tested the effects of immortalizing primary human mammary epithelial cells to mimic malignant progression, which we consider biologically relevant and meaningful [5]. Specifically, we measured a between-sites difference in median *wCDI* of 2%, whereas Kim et al. reported differences ranging from 5.8% to 12.8% for their primary strain 122L when treated with shRNA targeting p16 or cyclin D1. For recovery category, we reported a Cramér’s V of 0.094, and while Kim et al. did not report this figure, we computed a value of 0.242 based on their raw data, which is considered medium-to-large [19, 20]. Consequently, while our measurements of MCF-10A cells at Site A and Site B produced statistically significantly different results for *wCDI* and recovery category frequencies, the magnitudes of the effect sizes indicate that these differences may not be meaningful.

Based on this experiment, we conclude that the mechano-NPS results from one research group are highly replicable and reproducible when an identical experiment is performed by another group, even with differences in instrumentation hardware, environmental factors due to climate control, and different individuals performing the experiment. As such, we expect that our results here can be generalized to most other laboratories.

## Conclusions

Our work characterizing several sources of technical variability and assessing experimental reproducibility represents a novel kind of analysis regarding the use of microfluidic technologies for measuring cell mechanical properties. Increasingly complex and powerful devices can introduce greater opportunity for variability, and the repeatability of the measurements made with these technologies should be addressed accordingly. We investigated three aspects of the reproducibility of an experiment: (1) how technical variation between mechano-NPS devices might affect a measurement, (2) how the results of mechano-NPS measurements might vary due to the researcher processing the data, and (3) how mechano-NPS measurements might vary when performed by different research groups at different locations. A study such as this one provides a performance benchmark for other researchers who adopt a technology like mechano-NPS and sets expectations of technical variability to assess the reproducibility of experiments. Understanding and minimizing sources of technical variability is especially important because single-cell measurements are inherently sensitive to biological heterogeneity.

## Methods

### Device design

Design parameters for MCF-10A and AP-1060 devices followed designs from previously published work [5, 6]. Geometric features were chosen to optimize the signal-to-noise ratio of the microfluidic four-terminal measurement and apply a specific degree of strain to cells. See Fig 1B for the layout of each device type and Table 1 for each device’s geometric dimensions.

**Table 1:** Geometric dimensions of mechano-NPS devices.

Microfluidic feature	MCF-10A devices	AP-1060 devices
Channel height	22.3 μm	12.9 μm
Inline filter pore width	22 μm	20 μm
Pore width	22 μm	13 μm
Pore length	700 μm	800 μm
Node width	85 μm	85 μm
Node length	50 μm	50 μm
Contraction segment width	10.5 μm	7.0 μm
Contraction segment length	3000 μm	2000 μm
Recovery segment length	700 μm	290 μm
Targeted strain in contraction segment	0.30	0.35

### Device fabrication

The mechano-NPS channels were fabricated using standard soft lithography. Briefly, a negative-relief master was lithographically fabricated onto a polished silicon substrate using SU-8 epoxy photoresist (MicroChem) (SU-8 3025 for MCF-10A devices, SU-8 3010 for AP-1060 devices). Polydimethyl siloxane (PDMS) (Sylgard 184, Dow Corning) was mixed at a ratio of 9:1 pre-polymer base to curing agent, degassed with a vacuum desiccator, and then poured onto the negative relief masters. The PDMS was cured at 85°C (358 K) on a hotplate for 2 h, and a PDMS slab containing the embedded microfluidic channel was subsequently excised. The inlet and outlet ports were cored with a biopsy punch (Harris Uni-Core, Fisher Scientific).

Thin-film metal electrodes and contact pads were fabricated on a glass substrate. Briefly, standard photolithography was used to pattern Shipley 1813 photoresist (MicroChem) on the substrate. Electron-gun evaporation was then used to deposit a 75/250/250 Å titanium/platinum/gold thin film onto the patterned substrate, and photoresist liftoff was accomplished with immersion in acetone (JT Baker 9005-05 CMOS grade). For the MCF-10A devices, a gold wet etch solution (GOLD ETCHANT TFA, Transene Company) was drop-cast onto the area of electrodes crossing the microfluidic channel, exposing the platinum electrodes.

Mechano-NPS device fabrication was completed by treating the PDMS slab and glass substrate with pre-fabricated electrodes with oxygen plasma (Harrick Plasma, 450 mTorr (60 Pa), 30 W, 2 min). 20 μL of 2:1 methanol (ACS Grade, VWR BDH1135-4LG) to water (18.2 MΩ) was drop-cast onto the glass substrate to aid in alignment. The slab and substrate were then aligned, mated, and baked on a hotplate to evaporate the methanol-water mixture. The MCF-10A devices were baked at 85°C for 2 h, and the AP-1060 devices were baked at 125°C for 5 min.

### Cell culture

AP-1060 cells (DSMZ ACC 593), a gift from Dr. S. Kogan, University of California, San Francisco, CA, U.S.A., were cultured at 37°C with 5% CO_2_. They were initially seeded at a density of 1 x 10^6^ cells/mL in growth medium comprised of 70% Iscove’s modified Dulbecco’s medium (IMDM, Gibco 12440053), 20% fetal bovine serum (FBS, VWR 89510-186), 10% conditioned medium from cell line 5637 (ATCC HTB-9), and 1X Penicillin-Streptomycin (Gibco 15070063). Cells were passaged when suspension cultures reached a density of 2.5 x 10^6^ cells/mL. Conditioned medium from cell line 5637 was prepared by seeding 2.5 x 10^5^ cells in 10 mL of growth medium consisting of 90% RPMI-1640 (Corning 10-040-CV), 10% FBS, and 1X Penicillin-Streptomycin. Medium was changed after 24 h and collected after another 24 h. Before adding to AP-1060 growth medium, the conditioned medium was filtered using a 0.22 μm polyethersulfone filter (Millipore Sigma SLGPM33RS). For intra- and inter-user repeatability experiments, two samples of AP-1060 cells were treated with LatA (Abcam ab144290). LatA was reconstituted in ACS reagent grade ethyl alcohol (Sigma-Aldrich 459844) to a stock concentration of 2 mM, then aliquoted and kept frozen at –20°C until use. LatA was thawed and added to growth medium at a concentration of 2 μM. Cells were subsequently incubated in the LatA-supplemented growth medium for 30 min at 37°C with 5% CO_2_. After 30 min, LatA-treated cells were collected by centrifuging at 200 RCF for 5 min. Cells were washed once with 1X phosphate buffered saline (PBS) and centrifuged again for 5 min at 200 RCF before immediately being resuspended for mechano-NPS measurements (see below).

MCF-10A cells (ATCC CRL-10317) were cultured at 37°C and 5% CO_2_ in M87A medium containing cholera toxin and oxytocin at 0.5 ng/mL and 0.1 nM, respectively. Cells were passaged when adherent culture reached 75% confluence. After the fifth passage, cells were frozen at a concentration of 1 x 10^6^ cells/mL for use in reproducibility testing at Sites A and B. Upon thawing at Sites A and B, the cells were seeded at a density of 1 x 10^5^ cells/mL and medium was changed every 48h until they were passaged at 75% confluence, with the last medium change 24h prior to cell dissociation. Cell dissociation was accomplished by incubating cells in 0.25% trypsin-EDTA (Gibco, 25200056) at 37°C and 5% CO_2_ for 5 min, followed by trypsin neutralization with complete M87A medium (twice the volume of trypsin solution used). MCF-10A cells were collected by centrifuging at 200 RCF for 5 min, washed once with 1X PBS, then centrifuged again for 5 min at 200 RCF before immediately being resuspended for mechano-NPS measurements (see below).

### Mechano-NPS measurements

Suspension-culture AP-1060 cells were prepared for mechano-NPS by first transferring the cell suspension into microcentrifuge tubes and centrifuging for 5 min at 200 RCF. After aspirating the growth-medium supernatant, cells were washed with 1X PBS solution and centrifuged again for 5 min at 200 RCF. The PBS was aspirated, and the AP-1060 pellet was resuspended in 1X PBS supplemented with 2% FBS to reduce adhesion between cells and cell adhesion to PDMS. The cell density was diluted to 3 x 10^5^ cells/mL in 1X PBS supplemented with 2% FBS.

MCF-10A cells were prepared for mechano-NPS in a similar fashion. Cells were dissociated from the culture dish by incubating with 0.05% trypsin-EDTA (Gibco #25300062) for 10 min at 37°C, centrifuged for 5 min at 200 RCF, and resuspended in 1X PBS to a cell density of 1 x 10^5^ cells/mL.

To perform mechano-NPS measurements, diluted cell suspensions were aspirated into polytetrafluoroethylene tubing (1/32” (0.793 mm) ID, 1/16” (1.59 mm) OD, Cole-Parmer EW-06407-41) using a 1 mL slip-tip syringe (BD 309659) fitted with a 20-ga (0.91 mm) blunt-tip needle (Jensen Global JG20-0.5TX). For MCF-10A experiments, the tubing was inserted into a 25.4 mm (length) x 20-ga (0.91 mm, OD) 304 stainless steel connector (New England Small Tube Corp) which was then inserted into the inlet ports. For AP-1060 experiments, the tubing was directly inserted into PDMS inlet ports. The needle was then disconnected from the syringe and connected to an Elveflow OB1 microfluidic pressure controller. A nominal inlet pressure of 200 mbar (20 kPa) for MCF-10A experiments and 80 mbar (8 kPa) for AP-1060 experiments was applied to induce flow through the mechano-NPS device. A four-terminal current measurement was performed as previously described, using a DC potential of 2.5 V for MCF-10A experiments and 3 V for AP-1060 experiments, and measuring the current at a sample rate of 50 kHz [5, 7, 8, 21].

The instrumentation used to acquire the mechano-NPS data consisted of a custom-printed circuit board (PCB) to perform the four-terminal measurement, a benchtop power supply, a current preamplifier, and a PCIe DAQ to interface with a computer. The data was recorded using custom MATLAB software as previously published [5]. The same custom PCB was used at Site A and Site B, and the circuit diagram can be found in Kim et al. [5]. Site A used a Keysight E36311A power supply, a DL Instruments 1211 current preamplifier, and a National Instruments PCIe-6351 DAQ. Site B used a Hewlett-Packard/Agilent E3630A power supply, a Stanford Research Systems SR570 current preamplifier, and a National Instruments PCIe-6351 DAQ. The raw data was processed using custom MATLAB code as previously published to calculate the size and mechanical properties of measured cells [5–8].

### Data processing and analysis for mechano-NPS

Current data sampled at 50 kHz was first low-pass filtered using a 200-sample-wide rectangular moving average filter. The filtered signal was downsampled to 2.5 kHz, then detrended using asymmetric least squares smoothing [22]. The element-wise difference of the entire data vector was computed and thresholded according to user-supplied values; for example, for AP-1060 cell measurements, we used a normalized drop-change in current (relative to baseline) cutoff of 2 x 10^−4^ for pores and 1 x 10^−3^ for contraction segments. As a cell enters or exits a pore, it causes a step change in the current, which manifests as an extreme value in the first-order discrete time-difference in current. Thus, the threshold separates noise-related fluctuations from these step changes in current. As cells of different sizes generate step changes of varying amplitude, the threshold is set to a value specific to the signal-to-noise ratio of the data. By identifying these step changes, pulse and subpulse boundaries are established, allowing for calculation of subpulse amplitude (i.e., cell size) and duration (i.e., cell velocity). Mechanical parameters are computed as previously described [5, 6].

The user-dependent data processing pipeline is described in Fig 1C (code available at https://github.com/sohnlab/mechanoNPS_Li-et-al-2020/releases/tag/mNPS_2020). By finding peak locations in the first-order difference in current, a list of potential data “windows” where a pulse that might belong to a cell are generated. Many windows are automatically classified by the software as signal interference and discarded (e.g., if a subpulse is missing from the window). For the remaining windows, manual confirmation regarding whether the pulse belongs to a cell is necessary through a MATLAB CLI. Threshold values are adjusted as needed by the user before confirming to the program to extract information from the pulse in the window. The user can choose to let the program automatically compute threshold values based on the peak heights in the window or to manually adjust these values.

### Statistical methods and analysis

Statistical outliers, defined as more than 3 median absolute deviations from the sample median, were included in all statistical tests. Erroneous measurements were identified based on cell velocity by first excluding statistical outliers and then setting a cutoff of ± 4 median absolute deviations from the sample median velocity in either the reference pore or contraction segment. Erroneous measurements were excluded from all statistical tests and analyses unless otherwise stated.

Data for *wCDI* and recovery time constant were tested for normality using a Lilliefors test implemented in MATLAB R2020a. To evaluate differences in non-normal distributions, a Kruskal-Wallis test for non-parametric analysis of variance (ANOVA) across groups was implemented in MATLAB R2020a. For pairwise comparisons, a Wilcoxon rank sum test with a Tukey-Kramer method to correct the critical value (when applicable) was implemented in MATLAB R2020a. To determine if the probability distributions of *wCDI* and recovery time constant were equal, a two-sample Kolmogorov-Smirnov test with a Bonferroni correction (when applicable) was implemented in MATLAB R2020a. For recovery category data, the proportions of cells in each recovery category were analyzed with a Pearson’s Chi-squared test implemented in MATLAB R2020a.

Intra- and inter-user reliability analysis was performed on cell phenotype data resulting from each data processing observation of the same five raw data files. For all analyses of a given comparison, only cells that were saved in any observation within the comparison group were considered. The percent-agreement was calculated according to the decision to save or discard each observed cell, thus quantifying how often the observations agreed on whether to save or discard a cell (erroneous measurements were not excluded from this analysis). Fleiss’s kappa analysis was also performed according to the decision to save or discard each cell (using an implementation by Shah, 2020 in MATLAB R2020a), with the significance criterion of *α =* 0.05 referring to the threshold beyond which the agreement is statistically significantly better than chance (erroneous measurements were not excluded from this analysis) [23, 24]. Confidence intervals and p-values for Fleiss’s kappa analysis are reported in S3 Table. ICC analysis was performed on the data for *wCDI* and recovery time constant using the *irrNA* implementation in RStudio version 1.2 [25, 26], using a 2-way mixed-effects model to evaluate single-rater absolute agreement, with the null hypothesis that *ICC =* 0 evaluated at *α =* 0.05. S4 Table reports confidence intervals and p-values for ICC analysis, as well as the effect of excluding erroneous measurements in this analysis. Additionally, percent-agreement was calculated on the data for *wCDI* and recovery time constant, calculating the percent of cell events in which all observations found an equivalent value for the measured phenotype. Measured values were considered equivalent if the difference was within a tolerance of 1 x 10^−10^ multiplied by the minimum absolute value observed for the measured phenotype. S5 Table reports tolerance values used as well as the effect of excluding erroneous measurements on this analysis.

### Ethics statement

This human subjects study was conducted under an IRB-approved protocol (UC Berkeley Committee for Protection of Human Subjects, Protocol ID: 2020-11-13822), and all subjects provided written informed consent prior to taking part in the study.

## Supporting information

S1 Table Lilliefors tests for mechanical phenotyping data to determine distribution normality. The whole-cell deformability index *wCDI* (left) and recovery time constant *τ* (right) of AP-1060 cells measured on three different mechano-NPS devices were tested for normality using a Lilliefors test. A p-value less than 0.05 indicates a failure to reject the null hypothesis that the distribution of *wCDI* or recovery time constant for that device came from a normal distribution with an unspecified mean and standard deviation. *The test statistic exceeded the tabulated values in the MATLAB R2020a implementation of the Lilliefors test.
(PDF)

S2 Table Two-sample Kolmogorov-Smirnov tests to compare wCDI distributions from replicate devices. The distributions of *wCDI* for cells analyzed by seven different mechano-NPS devices were tested pairwise to determine if the data from each device came from equivalent distributions. A significance criterion of *α* = 0.05 was adjusted for multiple comparisons (21 pairwise comparisons) by a Bonferroni method. As such, any pairwise test with a p-value less than 0.0024 indicates that the data from the two devices tested are not sampled from the same distribution.
(PDF)

S3 Table Fleiss’s kappa analysis for the mechano-NPS data processing pipeline. Five subjects analyzed raw mechano-NPS data taken from AP-1060 cells; each subject processed each of the five blinded raw data files three different times using the mechano-NPS data processing software. The resulting list of cell measurements was analyzed using Fleiss’s kappa to quantify the inter- and intra-user agreement on whether to save or skip a given cell measurement. The kappa value is reported along with lower and upper bounds for the 95% confidence interval. A p-value less than 0.05 indicates a rejection of the null hypothesis that the observed agreement is accidental. This analysis was performed on all cell measurements, including those identified as erroneous. Number of cells found in each observation ranged from 49–82.
(PDF)

S4 Table Intra-class correlation of cell phenotype values using the mechano-NPS data processing pipeline. All subjects’ resulting measurements of the two cell phenotype values, *wCDI* and recovery time constant *τ*, were analyzed to quantify the inter- and intra-user consistency of the observed values. The intra-class correlation value (*ICC*) is reported along with lower and upper bounds for the 95% confidence interval. A p-value less then 0.05 indicates a rejection of the null hypothesis that *ICC =* 0. This analysis was performed both including and excluding the cell measurements that were identified as erroneous. Number of cells found in each observation ranged from 49–82.
(PDF)

S5 Table Percentage of equivalent measured cell phenotype values using the mechano-NPS data processing pipeline. All subjects’ resulting measurements of *wCDI* and *τ* were analyzed to quantify the percentage of cell events (“% agreement”) in which an equivalent phenotype value was found in all observations, within the reported tolerance. This analysis was performed both including and excluding the cell measurements that were identified as erroneous. Number of cells found in each observation ranged from 49–82.
(PDF)

S6 Table Frequencies of MCF-10A cell recovery categories measured at Site A and Site B. MCF-10A cells measured with mechano-NPS at Site A and Site B were classified according to whether they recovered from deformation instantaneously (*ΔT*_*r*_ = 0 ms), within a finite time window (0 < *ΔT*_*r*_ < 100 ms), or had prolonged recovery (*ΔT*_*r*_ > 100 ms).
(PDF)

S1 Fig Repeated analysis of raw data files using the mechano-NPS data processing software by multiple users. Five subjects analyzed raw mechano-NPS data taken from AP-1060 cells; each subject processed the raw data files three different times using the mechano-NPS data processing software. Heat maps show unique cell measurements as rows, and each subject’s repeated observations using the software as columns. Heat maps were generated using MATLAB R2020a with hierarchical clustering of rows and columns by Euclidean distance. The color scales show, for each observation, whether the cell measurement was saved or skipped (A), the measured *wCDI* value (B), and the measured recovery time constant (*τ*) value (C). Data is presented as originally returned after the data processing task was completed (i.e., before erroneous measurements were excluded). For (B) and (C), the color scale is limited to the non-extreme range of >0 and <2 for both *wCDI* and *τ*. For (B) and (C), cell measurements that were skipped in the given observation are colored gray, and the missing values were imputed for clustering using k-nearest-neighbors. The total number of cells measured across all observations was 398.
(TIF)
