# Supplementary material for: Evaluating sources of technical variability in the mechano-node-pore sensing pipeline and their effect on the reproducibility of single-cell mechanical phenotyping
Source: PLoS One. 2021 Oct 25;16(10):e0258982. doi: 10.1371/journal.pone.0258982 (PMC8544830; doi:10.1371/journal.pone.0258982)
Supplement: S1 Table — The whole-cell deformability index wCDI (left) and recovery time constant τ (right) of AP-1060 cells measured on three different mechano-NPS devices were tested for normality using a Lilliefors test. A p-value less than 0.05 indicates a failure to reject the null hypothesis that the distribution of wCDI or recovery time constant for that device came from a normal distribution with an unspecified mean and standard deviation. *The test statistic exceeded the tabulated values in the MATLAB R2020a implementation of the Lilliefors test. (PDF) [file pone.0258982.s001.pdf]

S1 Table. Lilliefors tests for mechanical phenotyping data to determine distribution normality.

wCDI

	p
Device 1	0.0029
Device 2	0.0011
Device 3	0.001*
Device 4	0.0047
Device 5	0.0278
Device 6	0.0446
Device 7	0.001*

Recovery time constant τ

	p
Device 1	0.0014
Device 2	0.3828
Device 3	0.001*
Device 4	0.001*
Device 5	0.001*
Device 6	0.1285
Device 7	0.0024

The whole-cell deformability index *wCDI* (left) and recovery time constant τ (right) of AP-1060 cells measured on seven different mechano-NPS devices were tested for normality using a Lilliefors test. A p-value less than 0.05 indicates a failure to reject the null hypothesis that the distribution of *wCDI* or recovery time constant for that device came from a normal distribution with an unspecified mean and standard deviation. *The test statistic exceeded the tabulated values in the MATLAB R2020a implementation of the Lilliefors test.
