# Supplementary material for: Evaluating sources of technical variability in the mechano-node-pore sensing pipeline and their effect on the reproducibility of single-cell mechanical phenotyping
Source: PLoS One. 2021 Oct 25;16(10):e0258982. doi: 10.1371/journal.pone.0258982 (PMC8544830; doi:10.1371/journal.pone.0258982)
Supplement: S2 Table — The distributions of wCDI for cells analyzed by seven different mechano-NPS devices were tested pairwise to determine if the data from each device came from equivalent distributions. A significance criterion of α = 0.05 was adjusted for multiple comparisons (21 pairwise comparisons) by a Bonferroni method. As such, any pairwise test with a p-value less than 0.0024 indicates that the data from the two devices tested are not sampled from the same distribution. (PDF) [file pone.0258982.s002.pdf]

S2 Table. Two-sample Kolmogorov-Smirnov tests to compare *wCDI* distributions from replicate devices.

	Dev. 1	Dev. 2	Dev. 3	Dev. 4	Dev. 5	Dev. 6	Dev. 7
Dev. 1							
Dev. 2	0.8612						
Dev. 3	0.1799	0.0420					
Dev. 4	0.0482	0.0054	0.8266				
Dev. 5	0.1178	0.0329	0.0012	0.0004			
Dev. 6	0.6037	0.2560	0.1290	0.0436	0.0210		
Dev. 7	0.0083	0.0241	0.8606	0.2614	< 0.0001	0.0423	

The distributions of *wCDI* for cells analyzed by seven different mechano-NPS devices were tested pairwise to determine if the data from each device came from equivalent distributions. A significance criterion of $\alpha = 0.05$ was adjusted for multiple comparisons (21 pairwise comparisons) by a Bonferroni method. As such, any pairwise test with a p-value less than 0.0024 indicates that the data from the two devices tested are not sampled from the same distribution.
