# Supplementary material for: Evaluating sources of technical variability in the mechano-node-pore sensing pipeline and their effect on the reproducibility of single-cell mechanical phenotyping
Source: PLoS One. 2021 Oct 25;16(10):e0258982. doi: 10.1371/journal.pone.0258982 (PMC8544830; doi:10.1371/journal.pone.0258982)
Supplement: S3 Table — Five subjects analyzed raw mechano-NPS data taken from AP-1060 cells; each subject processed each of the five blinded raw data files three different times using the mechano-NPS data processing software. The resulting list of cell measurements was analyzed using Fleiss’s kappa to quantify the inter- and intra-user agreement on whether to save or skip a given cell measurement. The kappa value is reported along with lower and upper bounds for the 95% confidence interval. A p-value less than 0.05 indicates a rejection of the null hypothesis that the observed agreement is accidental. This analysis was performed on all cell measurements, including those identified as erroneous. Number of cells found in each observation ranged from 49–82. (PDF) [file pone.0258982.s003.pdf]

S3 Table. Fleiss's kappa analysis for the mechano-NPS data processing pipeline.

Comparison	Subject(s)	kappa	lower bound	upper bound	*p*
intra-user	subject1	0.934	0.919	0.949	0
	subject2	0.853	0.837	0.868	0
	subject3	0.771	0.756	0.786	0
	subject4	0.562	0.547	0.578	0
	subject5	0.579	0.564	0.594	0
inter-user	sub1-sub2	0.595	0.573	0.616	0
	sub1-sub3	0.630	0.610	0.651	0
	sub1-sub4	0.306	0.288	0.325	0
	sub1-sub5	0.301	0.282	0.320	0
	sub2-sub3	0.493	0.474	0.513	0
	sub2-sub4	0.298	0.280	0.316	0
	sub2-sub5	0.292	0.274	0.310	0
	sub3-sub4	0.397	0.380	0.415	0
	sub3-sub5	0.362	0.344	0.380	0
	sub4-sub5	0.270	0.252	0.287	1.11e–15
	overall	0.389	0.383	0.394	0

Five subjects analyzed raw mechano-NPS data taken from AP-1060 cells; each subject processed each of the five blinded raw data files three different times using the mechano-NPS data processing software. The resulting list of cell measurements was analyzed using Fleiss's kappa to quantify the inter- and intra-user agreement on whether to save or skip a given cell measurement. The kappa value is reported along with lower and upper bounds for the 95% confidence interval. A p-value less than 0.05 indicates a rejection of the null hypothesis that the observed agreement is accidental. This analysis was performed on all cell measurements, including those identified as erroneous. Number of cells found in each observation ranged from 49–82.
