# Supplementary material for: Evaluating sources of technical variability in the mechano-node-pore sensing pipeline and their effect on the reproducibility of single-cell mechanical phenotyping
Source: PLoS One. 2021 Oct 25;16(10):e0258982. doi: 10.1371/journal.pone.0258982 (PMC8544830; doi:10.1371/journal.pone.0258982)
Supplement: S4 Table — All subjects’ resulting measurements of the two cell phenotype values, wCDI and recovery time constant τ, were analyzed to quantify the inter- and intra-user consistency of the observed values. The intra-class correlation value (ICC) is reported along with lower and upper bounds for the 95% confidence interval. A p-value less then 0.05 indicates a rejection of the null hypothesis that ICC = 0. This analysis was performed both including and excluding the cell measurements that were identified as erroneous. Number of cells found in each observation ranged from 49–82. (PDF) [file pone.0258982.s004.pdf]

S4 Table. Intra-class correlation of cell phenotype values using the mechano-NPS data processing pipeline.

			including all measurements				*excluding erroneous measurements*			
		**Subject(s)**	***ICC***	**lower bound**	**upper bound**	***p***	***ICC***	**lower bound**	**upper bound**	***p***
wCDI	intra-user	subject1	1.00	1.00	1.00	0	1.00	1.00	1.00	0
		subject2	1.00	1.00	1.00	0	1.00	1.00	1.00	0
		subject3	0.46	0.40	0.52	0	1.00	1.00	1.00	0
		subject4	-0.02	-0.08	0.05	0.673	1.00	1.00	1.00	0
		subject5	1.00	1.00	1.00	0	1.00	0.99	1.00	0
	inter-user	sub1-sub2	1.00	1.00	1.00	0	1.00	1.00	1.00	0
		sub1-sub3	-0.01	-0.12	0.09	0.596	0.96	0.95	0.97	0
		sub1-sub4	0.16	0.05	0.26	0.002	1.00	0.97	1.00	0
		sub1-sub5	0.00	-0.11	0.11	0.473	1.00	1.00	1.00	0
		sub2-sub3	-0.03	-0.13	0.08	0.684	0.97	0.96	0.98	0
		sub2-sub4	0.18	0.08	0.28	4.20e-4	1.00	1.00	1.00	0
		sub2-sub5	0.02	-0.09	0.13	0.368	1.00	1.00	1.00	0
		sub3-sub4	-0.02	-0.13	0.08	0.674	0.97	0.97	0.98	0
		sub3-sub5	0.70	0.64	0.75	0	0.97	0.96	0.97	0
		sub4-sub5	0.01	-0.10	0.11	0.459	1.00	1.00	1.00	0
		overall	0.10	0.06	0.14	1.01e-7	0.99	0.98	0.99	0
τ	intra-user	subject1	0.99	0.98	0.99	0	0.99	0.98	0.99	0
		subject2	0.92	0.90	0.93	0	0.92	0.90	0.93	0
		subject3	1.00	1.00	1.00	0	1.00	1.00	1.00	0
		subject4	0.99	0.99	0.99	0	0.95	0.94	0.96	0
		subject5	0.96	0.95	0.97	0	0.96	0.95	0.97	0
	inter-user	sub1-sub2	0.98	0.97	0.98	0	0.98	0.97	0.98	0
		sub1-sub3	0.83	0.79	0.86	0	0.82	0.79	0.86	0
		sub1-sub4	0.99	0.98	0.99	0	0.91	0.89	0.92	0
		sub1-sub5	0.90	0.88	0.92	0	0.90	0.88	0.92	0
		sub2-sub3	0.92	0.90	0.94	0	0.92	0.90	0.94	0
		sub2-sub4	0.99	0.99	1.00	0	0.97	0.96	0.97	0
		sub2-sub5	0.95	0.94	0.96	0	0.95	0.94	0.96	0
		sub3-sub4	0.99	0.99	0.99	0	0.95	0.94	0.96	0
		sub3-sub5	0.99	0.98	0.99	0	0.99	0.98	0.99	0
		sub4-sub5	0.99	0.99	0.99	0	0.97	0.97	0.98	0
		overall	0.98	0.97	0.98	0	0.94	0.93	0.95	0

All subjects' resulting measurements of the two cell phenotype values, *wCDI* and recovery time constant τ, were analyzed to quantify the inter- and intra-user consistency of the observed values.

The intra-class correlation value (*ICC*) is reported along with lower and upper bounds for the 95% confidence interval. A p-value less then 0.05 indicates a rejection of the null hypothesis that $ICC = 0$. This analysis was performed both including and excluding the cell measurements that were identified as erroneous. Number of cells found in each observation ranged from 49–82.
