# Supplementary material for: Evaluating sources of technical variability in the mechano-node-pore sensing pipeline and their effect on the reproducibility of single-cell mechanical phenotyping
Source: PLoS One. 2021 Oct 25;16(10):e0258982. doi: 10.1371/journal.pone.0258982 (PMC8544830; doi:10.1371/journal.pone.0258982)
Supplement: S5 Table — All subjects’ resulting measurements of wCDI and τ were analyzed to quantify the percentage of cell events (“% agreement”) in which an equivalent phenotype value was found in all observations, within the reported tolerance. This analysis was performed both including and excluding the cell measurements that were identified as erroneous. Number of cells found in each observation ranged from 49–82. (PDF) [file pone.0258982.s005.pdf]

S5 Table. Percentage of equivalent measured cell phenotype values using the mechano-NPS data processing pipeline.

Variable	Tolerance	Comparison	Subject(s)	% agreement, including all measurements	% agreement, excluding erroneous measurements
wCDI	4.30e–11	intra-user	subject1	88.9	88.9
			subject2	88.6	88.9
			subject3	87.7	88.7
			subject4	77.8	79.5
			subject5	65.1	66.4
		inter-user	sub1-sub2	75.5	75.8
			sub1-sub3	78.8	78.8
			sub1-sub4	73.3	74.9
			sub1-sub5	61.1	62.4
			sub2-sub3	81.7	81.9
			sub2-sub4	79.9	81.8
			sub2-sub5	67.3	68.8
			sub3-sub4	82.8	85.1
			sub3-sub5	69.3	71.2
			sub4-sub5	65.1	67.8
			overall	54.8	57.2
τ	5.80e–9	intra-user	subject1	84.8	84.8
			subject2	88.1	88.3
			subject3	87.7	88.7
			subject4	77.8	79.5
			subject5	60.5	61.7
		inter-user	sub1-sub2	73.1	73.3
			sub1-sub3	75.6	75.6
			sub1-sub4	69.6	71.1
			sub1-sub5	57.4	58.6
			sub2-sub3	80.6	80.8
			sub2-sub4	78.1	79.9
			sub2-sub5	64.1	65.6
			sub3-sub4	82.2	84.6
			sub3-sub5	65.5	67.4
			sub4-sub5	61.8	64.4
			overall	52.3	54.6

All subjects' resulting measurements of *wCDI* and τ were analyzed to quantify the percentage of cell events ("% agreement") in which an equivalent phenotype value was found in all observations, within the reported tolerance. This analysis was performed both including and excluding the cell

measurements that were identified as erroneous. Number of cells found in each observation ranged from 49–82.
