# Supplementary material for: Evaluating sources of technical variability in the mechano-node-pore sensing pipeline and their effect on the reproducibility of single-cell mechanical phenotyping
Source: PLoS One. 2021 Oct 25;16(10):e0258982. doi: 10.1371/journal.pone.0258982 (PMC8544830; doi:10.1371/journal.pone.0258982)
Supplement: S6 Table — MCF-10A cells measured with mechano-NPS at Site A and Site B were classified according to whether they recovered from deformation instantaneously (ΔTr = 0 ms), within a finite time window (0 < ΔTr < 100 ms), or had prolonged recovery (ΔTr > 100 ms). (PDF) [file pone.0258982.s006.pdf]

**S6 Table. Frequencies of MCF-10A cell recovery categories measured at Site A and Site B.**

| Recovery category | Time range | Number of recovered cells (% recovered cells) | |
|---|---|---|---|
| | | Site A | Site B |
| Instantaneous | $\Delta T_r = 0$ ms | 1519 (77.5) | 404 (64.7) |
| Finite | $0 < \Delta T_r < 100$ ms | 262 (13.4) | 149 (23.8) |
| Prolonged | $\Delta T_r > 100$ ms | 179 (9.1) | 72 (11.5) |

MCF-10A cells measured with mechano-NPS at Site A and Site B were classified according to whether they recovered from deformation instantaneously ($\Delta T_r = 0$ ms), within a finite time window ($0 < \Delta T_r < 100$ ms), or had prolonged recovery ($\Delta T_r > 100$ ms).
