# Supplementary figures and images for: Evaluating sources of technical variability in the mechano-node-pore sensing pipeline and their effect on the reproducibility of single-cell mechanical phenotyping
Source: PLoS One. 2021 Oct 25;16(10):e0258982. doi: 10.1371/journal.pone.0258982 (PMC8544830; doi:10.1371/journal.pone.0258982)

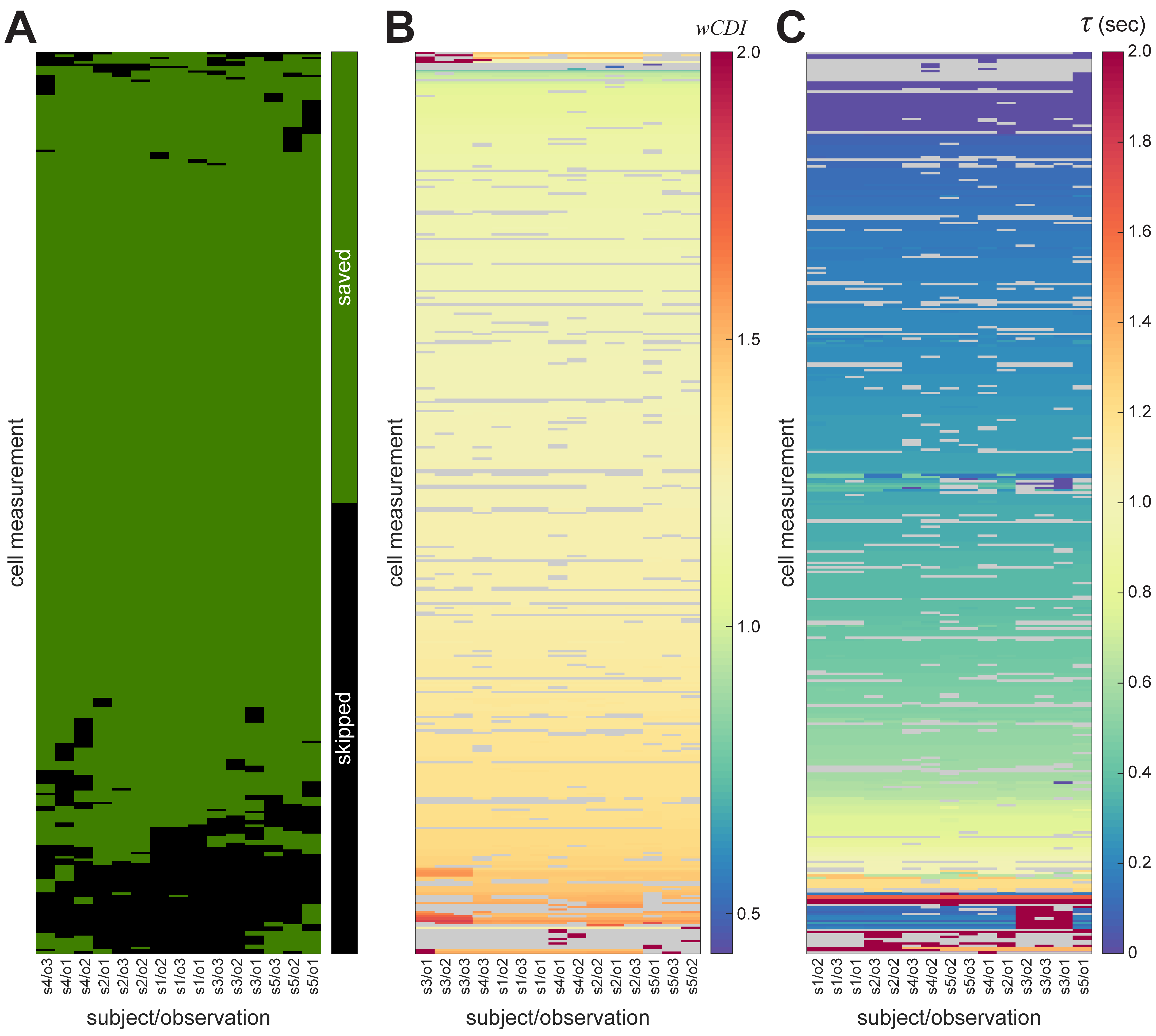

Supplement: S1 Fig — Five subjects analyzed raw mechano-NPS data taken from AP-1060 cells; each subject processed the raw data files three different times using the mechano-NPS data processing software. Heat maps show unique cell measurements as rows, and each subject’s repeated observations using the software as columns. Heat maps were generated using MATLAB R2020a with hierarchical clustering of rows and columns by Euclidean distance. The color scales show, for each observation, whether the cell measurement was saved or skipped (A), the measured wCDI value (B), and the measured recovery time constant (τ) value (C). Data is presented as originally returned after the data processing task was completed (i.e., before erroneous measurements were excluded). For (B) and (C), the color scale is limited to the non-extreme range of >0 and <2 for both wCDI and τ. For (B) and (C), cell measurements that were skipped in the given observation are colored gray, and the missing values were imputed for clustering using k-nearest-neighbors. The total number of cells measured across all observations was 398. (TIF) [file pone.0258982.s007.tif]
